# Modifiers of and Disparities in Palliative and Supportive Care Timing and Utilization among Neurosurgical Patients with Malignant Central Nervous System Tumors

**DOI:** 10.3390/cancers14102567

**Published:** 2022-05-23

**Authors:** Michael Chuwei Jin, Gary Hsin, John Ratliff, Reena Thomas, Corinna Clio Zygourakis, Gordon Li, Adela Wu

**Affiliations:** 1 Department of Neurosurgery, Stanford Health Care, Stanford, CA 94304, USA; mjin2@stanford.edu (M.C.J.); jratliff@stanford.edu (J.R.); corinnaz@stanford.edu (C.C.Z.); gordonli@stanford.edu (G.L.); 2 Department of Extended Care and Palliative Medicine Service, VA Palo Alto Health Care System, Palo Alto, CA 94304, USA; garyhsin@stanford.edu; 3 Department of Neurology and Neurological Sciences, Stanford Health Care, Stanford, CA 94304, USA; reenat@stanford.edu

**Keywords:** health inequities, brain cancer, spinal tumor, advanced cancer, racial diversity, palliative care, home health

## Abstract

**Simple Summary:**

Guidelines recommend early initiation of palliative care (PC) for patients with advanced cancers. Central nervous system (CNS) malignancies pose particular challenges for patients, who benefit from supportive care services such as PC, home health, and social work support. We analyze a cohort of privately insured patients with malignant brain or spinal tumors from the Optum Clinformatics Datamart Database to investigate health disparities in supportive care service access and utilization. We introduce a novel construct, “provider patient racial diversity index” (provider pRDI), the proportion of non-white minority patients a provider encounters to approximate a provider’s patient demographics and suggest a provider’s exposure to diversity. Our manuscript adds to existing literature on patient-level health disparities and provides a platform for future research focused on provider-level quality improvement interventions for utilization of supportive care services.

**Abstract:**

Patients with primary or secondary central nervous system (CNS) malignancies benefit from utilization of palliative care (PC) in addition to other supportive services, such as home health and social work. Guidelines propose early initiation of PC for patients with advanced cancers. We analyzed a cohort of privately insured patients with malignant brain or spinal tumors derived from the Optum Clinformatics Datamart Database to investigate health disparities in access to and utilization of supportive services. We introduce a novel construct, “provider patient racial diversity index” (provider pRDI), which is a measure of the proportion of non-white minority patients a provider encounters to approximate a provider’s patient demographics and suggest a provider’s cultural sensitivity and exposure to diversity. Our analysis demonstrates low rates of PC, home health, and social work services among racial minority patients. Notably, Hispanic patients had low likelihood of engaging with all three categories of supportive services. However, patients who saw providers categorized into high provider pRDI (categories II and III) were increasingly more likely to interface with supportive care services and at an earlier point in their disease courses. This study suggests that prospective studies that examine potential interventions at the provider level, including diversity training, are needed.

## 1. Introduction

Palliative care (PC) is defined as a critical service with the purpose of alleviating serious health-related suffering [1]. Social work services and home health agency services provide much-needed support beyond PC for patients with difficult cancer diagnoses and their families [2,3,4,5]. The American Society of Clinical Oncology (ASCO) recommends that patients with advanced cancer receive interdisciplinary and dedicated PC concurrent with active cancer care early in their disease courses [6]. According to ASCO, advanced cancer is defined as late-stage, distant metastases, or life-limiting with a prognosis of 6–24 months [6]. Usage of PC and related supportive services, such as home health and social work support, contributes to high-quality oncologic care.

Central nervous system (CNS) malignancies pose particular challenges for patients. Metastatic cancers may spread to the brain or spine, impacting function and quality of life. Glioblastoma (GBM) and other high-grade gliomas, such as astrocytoma or gliosarcoma, are commonly diagnosed primary brain malignancies in adults. While high-grade gliomas such as WHO Grade III anaplastic astrocytoma carry an approximate median survival time of 2–5 years, GBM, in particular, has a poor prognosis with a median overall survival of 16–21 months [7,8,9]. Historically, patients with brain metastases were precluded from participating in clinical trials due to presumed poor prognosis [10].

Despite national guidelines and recommendations, there are disparities in access to PC and other valuable supportive services among patients with advanced cancer, including those diagnosed with CNS malignancies. Retrospective studies on advanced cancers have identified racial minority background as a marker of poorer healthcare utilization and outcomes [11,12]. A recent national study also examined outcomes at the facility level by comparing minority-serving hospitals, with higher proportions of Black and Hispanic patients, to non-minority serving hospitals [13]. Unfortunately, minority-serving hospitals were significantly less likely to refer minority patients with metastatic cancer to PC, highlighting systemic problems underlying racial disparities [13].

We explore potential areas of healthcare quality improvement by creating a novel variable, “provider patient racial diversity index” (provider pRDI), which is defined by the proportion of non-white minority patients seen by a provider. The variable not only acts as a proxy for a provider’s practice and locale demographics but may offer insight into the level of cultural sensitivity of providers who demonstrate higher provider pRDI. We relied on the Optum Clinformatics Datamart Database (Optum) to construct a cohort of privately insured neurosurgical patients from all backgrounds who were diagnosed with a malignant CNS tumor. We aimed to (1) identify key modifiers of referral to and utilization of PC and supportive services, such as home health and social work; (2) investigate the impact of provider pRDI on supportive care utilization and referral timing; and (3) provide context for future necessary studies on healthcare inequities and potential provider-level quality improvement initiatives.

## 2. Materials and Methods

### 2.1. Data Source

All data used in this study was derived from Optum 2003–2021, which we have previously described and covers the healthcare claims of over 100 million enrollees. It includes the longitudinal healthcare service claims billed by providers in both inpatient and outpatient settings, which can be queried by provider class, setting, service type (as categorized by the Current Procedural Terminology (CPT) system), and diagnosis (as indicated by the International Classification of Diseases (ICD) system). All services were linked to encrypted provider and enrollee identifiers. This study was approved by our Institutional Review Board (#62056).

### 2.2. Cohort Design

All patients with at least one neurosurgery encounter (defined as a billed claim by a neurosurgeon) with a diagnosis of a malignant primary or secondary CNS tumor and no prior evidence of PC (defined as a billed claim by palliative or hospice care) were included in our study. The index diagnosis date was defined as the first qualifying tumor diagnosis code. At least 30 days of pre-index lookback, which was used for canvassing documented medical comorbidities, was required for study inclusion. Comorbidities were included based on the Elixhauser comorbidity index [14]. Other medical covariates included tumor etiology (primary versus metastatic) and receipt of surgery during the period of follow-up. Supportive care services were defined based on claims filed PC or hospice care providers. Similarly, social work services and home health services were identified based on provider categorization on each individual claim.

Patient-level demographics such as age, sex, and race were included in all analyses. Additionally, healthcare plans were categorized as Health Maintenance Organization (HMO), Exclusive Provider Organization (EPO), indemnity (IND), other (OTH), point-of-service (POS). We further sought to understand markers of physician exposure to patient diversity and overall cultural sensitivity. To do so, we defined a novel metric termed provider pRDI, which categorizes healthcare providers as category I, II, or III where increasing indices correlate with increasing exposure to patients of minority races. To estimate provider pRDI, we extracted all inpatient and outpatient services billed by each anonymized provider and mapped them to anonymized patients. From this, we estimated the fraction of patients served by each provider that were of a minority race to which a priori defined thresholds were applied. Specifically, providers whose patient population were less than 30%, between 30% and 49%, and over 50% minority race were termed “category I”, “category II”, and “category III” providers, respectively.

### 2.3. Statistical Analysis

The primary outcomes-of-interest were timing of supportive care services relative to initial tumor diagnosis and to documented death date. Other outcomes evaluated included incidence of supportive services as well as total utilization of these services based on healthcare spending. Multivariable mixed effects Cox, logistic, and linear regression were used to evaluate incidence of care initiation, incidence of utilization, and cumulative costs, respectively. Propensity score matching was used to generate matched cohorts balanced for demographics and comorbidities and conducted in a 1:1:1 approach using a greedy matching algorithm. Covariate balance was evaluated by computing standardized mean differences (SMD). All analyses were conducted in The R Project for Statistical Computing, version 4.0.0 (R Core Team, Indianapolis, IN, USA) and GraphPad Prism 8 (GraphPad Software, San Diego, CA, USA)

## 3. Results

**Figure 1:**
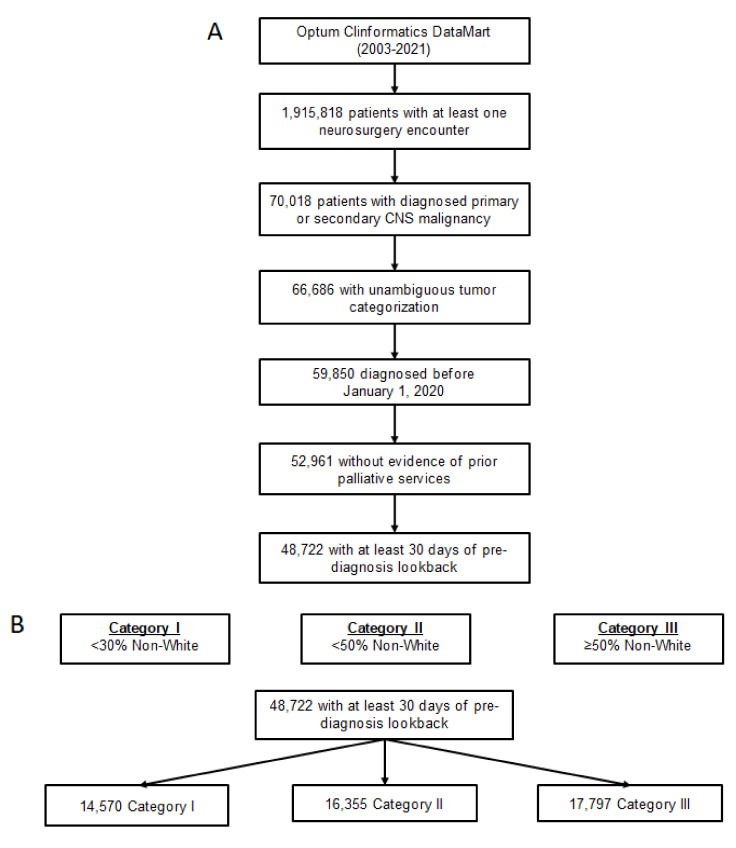
**Cohort flowchart and provider patient racial diversity index**. (**A**) Flowchart of neurosurgical patients with central nervous system (CNS) malignancies included for analysis, derived from Optum 2003–2021. (**B**) Designation and breakdown of provider patient racial diversity index categories.

In total, 48,722 patients met all inclusion criteria (Figure 1). Full cohort characteristics are detailed in Table 1. Notably, the number of secondary malignancies (*N* = 23,554) was nearly equal to the number of primary malignancies (*N* = 25,168). Additionally, the distribution of provider pRDI was nearly even, with 14,570 patients qualifying under category I (29.9%), 16,355 under category II (33.6%), and 17,797 under category III (36.5%).

Overall, 12,805 patients received at least one PC referral (26.3%), 3,612 patients received social work services (7.4%), and 11,488 patients received home health services (23.6%) during the period of post-diagnosis follow-up. Among those that did receive PC, median time to PC initiation was 96 days. This was lower for those with newly diagnosed secondary malignancies (86 days vs. 117 days, *p* < 0.001).

On multivariable regression analysis of time to service initiation, Hispanic race was associated with decreased initiation of PC (versus white, OR 0.882, 95% CI 0.813 to 0.958, Table 2). Similarly, Hispanic and Asian race were associated with decreased initiation of home health services while all minority races were associated with reduced initiation of social work services. In contrast, higher provider pRDI was associated with higher incidence of initiating palliative, home health, and social work services (II vs. I, OR 1.347, 95% CI 1.271 to 1.429; III vs. I, OR 1.478, 95% CI 1.396 to 1.566).

Regarding total healthcare spending, patient race did not impact cumulative utilization of palliative, home health, or social work services (Table 3). However, those patients qualifying under category II had significantly higher PC spending (vs. I, B = 276.364, 95% CI 138.550 to 414.179) while those qualifying under category III had significantly higher spending on both PC (vs. I, B = 439.061, 95% CI 301.514 to 576.608) and home health services (vs. I, B = 849.411, 95% CI 393.651 to 1305.171).

We also analyzed impact of gender and insurance type on incidence of supportive care services. In general, gender had no impact on service initiation, and male gender was only significantly associated with lower likelihood of referral to social work (vs. female, OR 0.850, Table 2). For the most part, private insurance plans were related to greater rates of initiation of supportive care services, although the results for home health and social work referral were variable (Table 2).

**Figure 2:**
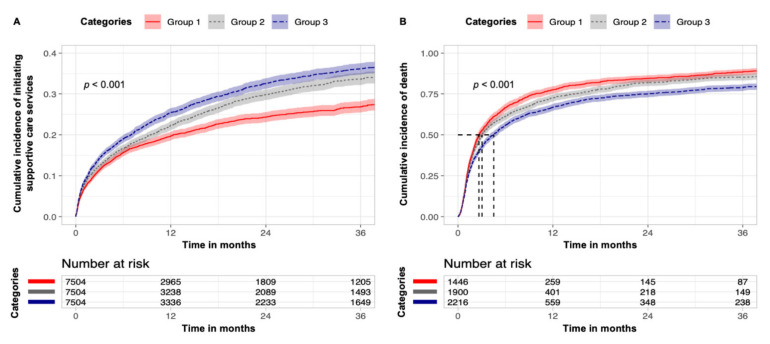
**Timing of initiation of supportive care services**. (**A**) Cumulative incidence of initiating supportive care services over time stratified by provider patient racial diversity index (provider pRDI) categories for the matched cohort. (**B**) Cumulative incidence of death following supportive care service initiation over time stratified by provider pRDI.

**Figure 3:**
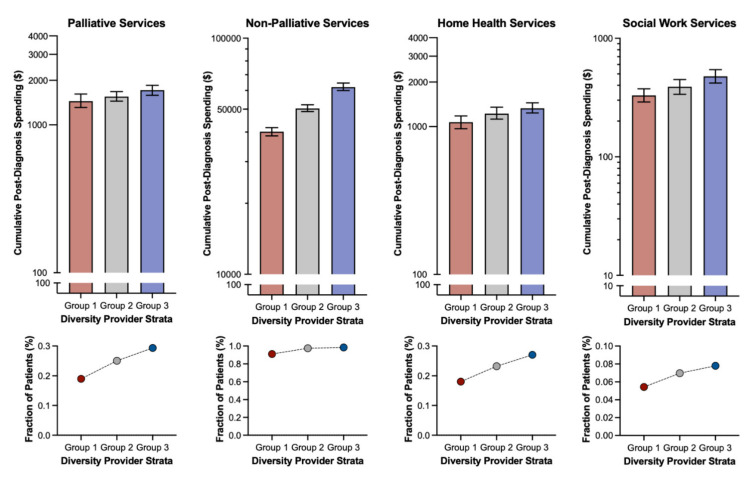
**Effect of provider patient racial diversity index (provider pRDI)**: effect of provider pRDI on spending for palliative, non-palliative, home health, and social work services in the matched cohort.

After matching, we aimed for covariate balance, particularly those associated with demographics and plan type (Table 1). Comparing these matched cohorts stratified by provider pRDI, patients classified within higher categories had significantly higher incidence of initiating supportive care services (Figure 2A, *p* < 0.001). Furthermore, incidence of death following initiation of these services was significantly lower among patients of higher categories, indicating earlier supportive care service involvement (Figure 2B, *p* < 0.001). Comparing overall cumulative use of palliative, home health, and social work services, patients classified under higher provider pRDI categories demonstrated significantly higher utilization. Comparing palliative, non-palliative, home health, and social work services, increasing provider pRDI was associated with monotonic increases in both spending and prevalence of utilization (Figure 3).

## 4. Discussion

Our final cohort included 48,722 privately insured neurosurgical patients with diagnoses of primary and secondary CNS malignancies. Our analysis demonstrates statistically significant low rates of PC, home health, and social work services among patients of racial minority groups, even though, over time, all patients had greater likelihood of referral to these services. Hispanic patients had low likelihood of engaging with all three categories of supportive care services. Black, Asian, and Hispanic patients all had significantly lower utilization of social work services. However, patients who saw providers categorized into high provider pRDI (categories II and III) were increasingly more likely to interface with supportive care services and at an earlier point in their disease courses.

### 4.1. Racial Disparities in Treatment and Surgical Outcomes

Pervasive racial disparities exist for patients with CNS malignancies in accessing high-quality care at specialized centers. Hospitals that receive high volumes of patients with CNS malignancies arguably have better postoperative outcomes, but a retrospective study revealed that Hispanic white patients with GBM compared to non-Hispanic white patients had significantly lower odds of receiving surgery at a high-volume center (OR 0.58, 95% CI 0.49–0.69, *p* < 0.001) [15].

Race appears to influence treatment options for patients with CNS malignancies as well. Out of 103,652 patients, non-Hispanic white patients had significantly higher rates of gross total surgical resection of their GBM (30.7%) as well as receipt of chemotherapy (65.8%) [16]. In addition, African American patients with metastatic spinal disease were less likely to receive surgery (OR 0.71, 95% CI 0.62–0.82, *p* < 0.001; RR 0.80, 95% CI 0.70–0.93) compared to white patients [17,18]. Race-based disparities were also apparent when comparing utilization of conventional external beam radiation versus more modern spinal stereotactic body radiation therapy for spinal metastases treatment; African American background was significantly more associated with the traditional radiation modality (adjusted OR 0.8, 95% CI 0.7–1.0) [19].

Racial background affects neurosurgical outcomes and post-operative disposition. Our cohort indicates that patients from minority backgrounds, namely Hispanic and Asian, are not referred to home health services as often as their Caucasian counterparts. Such a finding is reflected in published neurosurgical literature as well. For patients who underwent craniotomies for brain tumor resection, being from a Black background increased the risk of non-home disposition and extended length of stay by 6.9% and 6.5%, respectively, compared to white patients [20]. A similar phenomenon was seen in cohorts of Black patients who had undergone surgery for spine metastases, where the odds of non-home discharge were significantly higher than for white patients (adjusted OR 2.24, 95% CI 1.28–3.92, *p* = 0.005; OR 1.19, 95% CI 1.05–1.35, *p* = 0.007) [17,21]. Patients’ racial backgrounds impact their surgical care and postoperative outcomes, such as non-routine dispositions other than the ideal home discharge with appropriate home health and supportive services.

### 4.2. Racial Disparities in Palliative and Supportive Care

Patients with advanced cancer experience disparities in access to various supportive care services, such as PC. Minorities with advanced cancer expressed greater needs for support, including psychological, financial, social, and daily living aid [22]. These differences persisted for up to 12 months of follow-up for the cohort of patients with newly diagnosed advanced lung cancer [22]. Even though African American patients with advanced cancer perceived greater needs for hospices, they were among the lowest utilizers of such services [23,24]. Beyond hospice services, patients of racial minorities also had higher symptom burden—depressed mood, pain, and fatigue—by the time of referral to an institution’s Supportive Care Center [25]. As for patients with CNS malignancies, such as brain metastases, non-white patients were less likely to receive PC [26]. There were no recent and relevant publications with data on minority patients who suffer from spinal tumors.

### 4.3. Provider Influences on Quality of Healthcare for Minority Groups

An opportunity for systems-level modification is the influence of *providers* on the quality of healthcare delivered for minority patients. Providers from racial minority backgrounds were more likely to care for underserved, minority patients [27]. By focusing on providers and the diversity of their patient populations, we sought to not only use provider pRDI as a proxy of the practice’s demographic diversity but also allude to its potential utility of assessing a provider’s individual cultural sensitivity. In our cohort, the level of provider pRDI shows a clear correlation with increased utilization, spending, and earlier referral patterns of all supportive services: PC, social work, and home health.

Unfortunately, there are challenges in effective medical communication and delivering care for minority patients. First, language barriers prevent access to quality healthcare. A systematic review of 33 studies on patient–provider relationships where the patients’ primary language was not English demonstrated that the vast majority of studies reported favorable outcomes for language-concordant care and 9% of studies resulted in worse outcomes for language-discordant care [28]. Furthermore, not only do physicians communicate less effectively with minority patients, but these patients also express their needs less assertively than do white patients [29]. Such a phenomenon may predispose minority patients to receive fewer recommendations for care, highlighting potential underlying provider biases [29,30]. One study offers an alternative finding, where unconscious racial biases were not necessarily associated with clinical decision making in the acute surgical care setting, even though such biases were present in most surveyed physician respondents [31].

Racial concordance between patient and provider is an important concept that has been previously studied and indicates higher patient satisfaction and quality of delivered healthcare. For example, Black patients rated their Black doctors as “excellent” (adjusted OR 2.40, 95% CI 1.55–3.72) and reported receiving all necessary and recommended medical care in the past year (adjusted OR 2.94, 95% CI 1.10–7.87) [32]. A similar effect on patient satisfaction was reported by Hispanic patients who saw Hispanic physicians (adjusted OR 1.74, 95% CI 1.01–2.99) [32].

However, in situations where racial concordance may not be obtained, it is still critical to address and encourage cultural sensitivity among providers. In one survey of surgical oncologists, 71% of respondents reported seeing patients from six or more racial minority groups, although only 58% of providers received specific cultural diversity training [33]. Those who completed such training scored higher on the Cultural Competence Assessment than surgeons not exposed to diversity training (10.56 versus 9.82, *p* < 0.001) [33]. Our study’s findings regarding provider pRDI suggest the importance of exposure to racial diversity in patient populations and, by extension, cultural sensitivity in providing quality care for racial minority patients. Future prospective studies to examine the association between provider pRDI and the utilization and timing of healthcare resources, such as PC and other supportive care services, would need to be conducted.

Most published research on the topics of racial disparities in oncology and palliative care were conducted within the American healthcare system, with one Australian study assessing rates of chemotherapy administration in culturally and linguistically diverse patient populations [34]. While the aforementioned retrospective analysis determined no differences in adjuvant chemotherapy use, additional studies are warranted to characterize the prevalence and contributing factors of health inequities in diverse healthcare systems beyond the United States [34].

Even with many publications identifying and acknowledging the reality of racial disparities, more research is required to elucidate potential root causes and methods of critically assessing health inequities. For instance, it has been demonstrated that the quality of healthcare minority patients receive is influenced by *where* these patients access care [13,35,36]. Beyond characterizing hospital facilities, health disparity research may also benefit from a focus on healthcare providers themselves.

Limitations of our study include those inherent to retrospective studies based on nationwide claims databases. These include selection bias and missing or miscoded data and variables. The Optum database itself includes only patients who are privately insured, resulting in a more homogeneous sampling for the patient cohort in question. All diagnoses and claims were also identified based on standard coding systems, such as ICD, and cannot be subject to further verification for accuracy. We introduce the novel construct—provider pRDI—in our study, as the Optum database does not include provider details, such as experience level, or granular geographic and demographic details, such as racial makeup, income levels or descriptors of households, of the patient population in a certain locale. In addition, it is not yet known whether our study findings are generalizable to healthcare systems beyond the United States. Strengths of our study include the number of analyzed patients as well as the longitudinal aspect of the analyses from diagnosis of CNS malignancy until death in terms of investigating timing of PC and other supportive services. Future studies can take myriad directions: the impact of specific socioeconomic or provider-level factors on supportive care utilization and referral patterns; the influence of provider pRDI on other aspects of high-quality end-of-life care, such as shared care plans and advance care planning; or the longitudinal effects of cultural sensitivity training for providers on provider pRDI and utilization of supportive care services [37].

## 5. Conclusions

Patients suffering from malignancies of the central nervous system are less likely to receive palliative and other supportive services in a timely manner when they are of racial minority backgrounds. Such an effect is mitigated when these patients encounter at least one provider who scores highly on the provider patient racial diversity index—a measure of the proportion of non-white patients seen by the said provider. Patients seen by providers who encounter a more diverse patient population also receive supportive care services earlier in their disease courses. Our study highlights not only patient-level healthcare disparities in access to and utilization of quality palliative and supportive healthcare but also the possibility and need for provider-level intervention.

## Figures and Tables

**Table 1:** Unmatched and matched cohort characteristics.

Unmatched Cohort Characteristics								
**Characteristic**	Category 1 (*N* = 14,570)		Category 2 (*N* = 16,355)		Category 3 (*N* = 17,797)		SMD (0 vs. 1)	SMD (0 vs. 1)
	*N*	*%*	*N*	*%*	*N*	*%*		
**Year of Diagnosis**	2013.77	4.77%	2011.01	4.47%	2011.83	4.52%	0.597	0.417
**Age at Diagnosis (years)**	61.31	16.2%	58.56	21.16%	60.83	14.83%	0.146	0.031
**Sex**							0.044	0.075
Female (ref)	7146	49%	8385	51.3%	9399	52.8%		
Male	7424	51%	7970	48.7%	8398	47.2%		
**Race**							0.105	0.582
White (ref)	12,635	86.7%	14,671	89.7%	11,156	62.7%		
Asian	284	1.9%	249	1.5%	874	4.9%		
Black	806	5.5%	812	5%	3453	19.4%		
Hispanic	845	5.8%	623	3.8%	2314	13%		
**Tumor Type**							0.498	0.564
Primary (ref)	10,128	69.5%	7466	45.6%	7574	42.6%		
Secondary	4442	30.5%	8889	54.4%	10,223	57.4%		
**Insurance Plan**							0.48	0.203
HMO	3668	25.2%	3136	19.2%	4205	23.6%		
EPO	602	4.1%	1208	7.4%	1351	7.6%		
IND	295	2%	461	2.8%	336	1.9%		
OTH	4213	28.9%	2175	13.3%	4155	23.3%		
POS	4549	31.2%	7568	46.3%	5767	32.4%		
PPO	1243	8.5%	1807	11%	1983	11.1%		
**Received Surgery Post-Diagnosis**	9271	63.6%	10,540	64.4%	11,861	66.6%	0.017	0.063
**Comorbidities**								
Congestive Heart Failure	116	0.8%	104	0.6%	175	1%	0.019	0.02
Cardiac Arrhythmia	342	2.3%	323	2%	404	2.3%	0.026	0.005
Valvular Disease	119	0.8%	110	0.7%	160	0.9%	0.017	0.009
Pulmonary Circulation Disorders	56	0.4%	34	0.2%	62	0.3%	0.032	0.006
Peripheral Vascular Disorders	168	1.2%	140	0.9%	227	1.3%	0.03	0.011
Hypertension Uncomplicated	1487	10.2%	1356	8.3%	2159	12.1%	0.066	0.061
Hypertension Complicated	88	0.6%	110	0.7%	196	1.1%	0.009	0.054
Paralysis	42	0.3%	29	0.2%	50	0.3%	0.023	0.001
Chronic Pulmonary Disease	511	3.5%	603	3.7%	759	4.3%	0.01	0.039
Diabetes Uncomplicated	605	4.2%	598	3.7%	1032	5.8%	0.026	0.076
Diabetes Complicated	186	1.3%	110	0.7%	264	1.5%	0.062	0.018
Hypothyroidism	380	2.6%	356	2.2%	445	2.5%	0.028	0.007
Renal Failure	144	1%	107	0.7%	212	1.2%	0.037	0.02
Liver Disease	91	0.6%	125	0.8%	163	0.9%	0.017	0.033
Peptic Ulcer Disease excluding bleeding	16	0.1%	13	0.1%	28	0.2%	0.01	0.013
AIDS/HIV	7	0%	11	0.1%	34	0.2%	0.008	0.041
Rheumatoid Arthritis/Collagen	131	0.9%	128	0.8%	165	0.9%	0.013	0.003
Coagulopathy	50	0.3%	70	0.4%	81	0.5%	0.014	0.018
Obesity	176	1.2%	113	0.7%	170	1%	0.053	0.024
Weight Loss	35	0.2%	71	0.4%	92	0.5%	0.033	0.045
Fluid and Electrolyte Disorders	137	0.9%	175	1.1%	242	1.4%	0.013	0.039
Blood Loss Anemia	10	0.1%	17	0.1%	31	0.2%	0.012	0.03
Deficiency Anemia	150	1%	185	1.1%	233	1.3%	0.01	0.026
Alcohol Abuse	25	0.2%	31	0.2%	33	0.2%	0.004	0.003
Drug Abuse	24	0.2%	19	0.1%	32	0.2%	0.013	0.004
Psychoses	58	0.4%	32	0.2%	56	0.3%	0.037	0.014
Depression	387	2.7%	375	2.3%	396	2.2%	0.023	0.028
**Matched Cohort Characteristics**								
**Characteristic**	Category 1 (*N* = 7504)		Category 2 (*N* = 7504)		Category 3 (*N* = 7504)		SMD (0 vs. 1)	SMD (0 vs. 1)
	*N*	*%*	*N*	*%*	*N*	*%*		
**Year of Diagnosis**	2011.19	4.55%	2010.99	4.62%	2011.36	4.52%	0.043	0.037
**Age at Diagnosis (years)**	62.52	15.22%	62.36	15.1%	61.71	15.42%	0.01	0.052
**Sex**							0.055	0.019
Female (ref)	3756	50.1%	3961	52.8%	3826	51%		
Male	3748	49.9%	3543	47.2%	3678	49%		
**Race**							<0.001	<0.001
White (ref)	7093	94.5%	7093	94.5%	7093	94.5%		
Asian	78	1%	78	1%	78	1%		
Black	189	2.5%	189	2.5%	189	2.5%		
Hispanic	144	1.9%	144	1.9%	144	1.9%		
**Tumor Type**							0.062	0.069
Primary (ref)	3470	46.2%	3237	43.1%	3213	42.8%		
Secondary	4034	53.8%	4267	56.9%	4291	57.2%		
**Insurance Plan**							<0.001	<0.001
HMO	2302	30.7%	2302	30.7%	2302	30.7%		
EPO	277	3.7%	277	3.7%	277	3.7%		
IND	213	2.8%	213	2.8%	213	2.8%		
OTH	1702	22.7%	1702	22.7%	1702	22.7%		
POS	2247	29.9%	2247	29.9%	2247	29.9%		
PPO	763	10.2%	763	10.2%	763	10.2%		
**Received Surgery Post-DX**	4548	60.6%	4742	63.2%	5129	68.4%	0.053	0.162
**Comorbidities**								
Congestive Heart Failure	62	0.8%	64	0.9%	88	1.2%	0.003	0.035
Cardiac Arrhythmia	173	2.3%	205	2.7%	222	3%	0.027	0.041
Valvular Disease	51	0.7%	59	0.8%	84	1.1%	0.012	0.047
Pulmonary Circulation Disorders	29	0.4%	17	0.2%	31	0.4%	0.029	0.004
Peripheral Vascular Disorders	101	1.3%	90	1.2%	119	1.6%	0.013	0.02
Hypertension Uncomplicated	759	10.1%	760	10.1%	1063	14.2%	<0.001	0.124
Hypertension Complicated	53	0.7%	72	1%	84	1.1%	0.028	0.043
Paralysis	20	0.3%	17	0.2%	27	0.4%	0.008	0.017
Chronic Pulmonary Disease	320	4.3%	369	4.9%	392	5.2%	0.031	0.045
Diabetes Uncomplicated	326	4.3%	355	4.7%	518	6.9%	0.019	0.111
Diabetes Complicated	60	0.8%	68	0.9%	112	1.5%	0.012	0.065
Hypothyroidism	186	2.5%	210	2.8%	210	2.8%	0.02	0.02
Renal Failure	75	1%	59	0.8%	97	1.3%	0.023	0.028
Liver Disease	54	0.7%	74	1%	81	1.1%	0.029	0.038
Peptic Ulcer Disease excluding bleeding	9	0.1%	8	0.1%	17	0.2%	0.004	0.026
AIDS/HIV	3	0%	6	0.1%	19	0.3%	0.016	0.056
Rheumatoid Arthritis/Collagen	59	0.8%	63	0.8%	83	1.1%	0.006	0.033
Coagulopathy	33	0.4%	34	0.5%	53	0.7%	0.002	0.035
Obesity	51	0.7%	57	0.8%	76	1%	0.009	0.036
Weight Loss	31	0.4%	38	0.5%	43	0.6%	0.014	0.023
Fluid and Electrolyte Disorders	86	1.1%	105	1.4%	131	1.7%	0.023	0.05
Blood Loss Anemia	8	0.1%	11	0.1%	16	0.2%	0.011	0.027
Deficiency Anemia	87	1.2%	117	1.6%	101	1.3%	0.035	0.017
Alcohol Abuse	13	0.2%	23	0.3%	19	0.3%	0.027	0.017
Drug Abuse	6	0.1%	8	0.1%	14	0.2%	0.009	0.029
Psychoses	17	0.2%	25	0.3%	31	0.4%	0.02	0.033
Depression	186	2.5%	228	3%	222	3%	0.034	0.03

**Table 2:** Mixed effects model evaluating incidence of supportive care service utilization.

Characteristic	Palliative Care		Home Health Services		Social Worker Services	
	OR	*p*-Value	OR	*p*-Value	OR	*p*-Value
**Year of Diagnosis**	**1.027**	**<0.001**	**0.935**	**<0.001**	**1.029**	**<0.001**
**Age at Diagnosis (years)**	**1.011**	**<0.001**	**1.005**	**<0.001**	**0.983**	**<0.001**
**Sex**						
Female (ref)						
Male	0.971	0.178	0.981	0.389	**0.850**	**<0.001**
**Race**						
White (ref)						
Asian	0.881	0.053	**0.842**	**0.013**	**0.599**	**<0.001**
Black	1.031	0.396	0.981	0.612	**0.756**	**<0.001**
Hispanic	**0.882**	**0.003**	**0.900**	**0.016**	**0.819**	**0.003**
**Tumor Type**						
Primary (ref)						
Secondary	**1.678**	**<0.001**	**1.398**	**<0.001**	**0.737**	**<0.001**
**Insurance Plan**						
HMO						
EPO	**1.587**	**<0.001**	**1.401**	**<0.001**	1.109	0.185
IND	**0.274**	**<0.001**	**0.299**	**<0.001**	**2.166**	**<0.001**
OTH	**2.197**	**<0.001**	**0.391**	**<0.001**	**0.822**	**0.003**
POS	**1.744**	**<0.001**	**1.582**	**<0.001**	**1.239**	**<0.001**
PPO	**1.656**	**<0.001**	**0.863**	**<0.001**	1.013	0.856
**Received Surgery Post-Diagnosis**	**1.772**	**<0.001**	**2.263**	**<0.001**	**1.458**	**<0.001**
**Provider pRDI Category**						
I						
II	**1.347**	**<0.001**	**1.268**	**<0.001**	**1.335**	**<0.001**
III	**1.478**	**<0.001**	**1.556**	**<0.001**	**1.498**	**<0.001**
**Comorbidities**						
Congestive Heart Failure	0.827	0.131	1.042	0.765	0.587	0.055
Cardiac Arrhythmia	0.951	0.505	0.881	0.153	0.840	0.249
Valvular Disease	1.000	0.999	**1.360**	**0.019**	1.476	0.056
Pulmonary Circulation Disorders	0.874	0.481	0.763	0.234	**1.802**	**0.033**
Peripheral Vascular Disorders	0.947	0.591	**0.739**	**0.020**	0.751	0.201
Hypertension Uncomplicated	0.964	0.344	0.987	0.763	**0.816**	**0.005**
Hypertension Complicated	1.065	0.613	0.842	0.262	0.583	0.058
Paralysis	0.906	0.672	1.426	0.112	1.452	0.207
Chronic Pulmonary Disease	1.011	0.851	1.003	0.957	0.861	0.164
Diabetes Uncomplicated	1.093	0.095	**1.150**	**0.020**	**1.302**	**0.004**
Diabetes Complicated	1.172	0.103	0.881	0.318	1.330	0.093
Hypothyroidism	1.066	0.353	1.075	0.346	1.093	0.438
Renal Failure	0.970	0.794	0.947	0.702	1.230	0.338
Liver Disease	1.127	0.301	1.210	0.122	0.800	0.315
Peptic Ulcer Disease excluding bleeding	1.692	0.063	1.132	0.709	1.388	0.506
AIDS/HIV	0.950	0.879	1.540	0.164	0.833	0.762
Rheumatoid Arthritis/Collagen	0.976	0.829	**1.327**	**0.016**	1.126	0.515
Coagulopathy	1.113	0.509	1.380	0.056	1.197	0.522
Obesity	0.989	0.918	1.019	0.885	**1.437**	**0.026**
Weight Loss	1.186	0.292	**1.533**	**0.010**	0.763	0.403
Fluid and Electrolyte Disorders	0.957	0.671	0.946	0.630	1.021	0.907
Blood Loss Anemia	0.640	0.154	0.913	0.787	0.453	0.280
Deficiency Anemia	1.009	0.923	1.180	0.137	0.945	0.753
Alcohol Abuse	0.736	0.238	0.891	0.672	0.830	0.649
Drug Abuse	0.981	0.945	1.077	0.803	0.469	0.152
Psychoses	1.331	0.131	0.744	0.244	**2.712**	**<0.001**
Depression	1.118	0.112	1.142	0.072	**2.775**	**<0.001**

**Table 3:** Mixed effects model evaluating supportive care service spending.

Characteristic	Palliative Care		Home Health Services		Social Worker Services	
	B	*p*-Value	B	*p*-Value	B	*p*-Value
**Year of Diagnosis**	−6.284	0.328	**−50.284**	**0.018**	−1.836	0.618
**Age at Diagnosis (years)**	**6.053**	**<0.001**	−3.555	0.537	1.07	0.283
**Sex**						
Female (ref)						
Male	**−208.974**	**<0.001**	−136.668	0.432	−20.919	0.487
**Race**						
White (ref)						
Asian	−71.749	0.648	−513.737	0.324	169.436	0.060
Black	104.263	0.242	−434.966	0.141	−87.706	0.086
Hispanic	83.091	0.407	−545.657	0.101	−51.849	0.367
**Tumor Type**						
Primary (ref)						
Secondary	**−177.758**	**0.001**	**−419.209**	**0.023**	−39.836	0.213
**Insurance Plan**						
HMO						
EPO	**−533.252**	**<0.001**	−140.992	0.720	**−185.904**	**0.006**
IND	**−1095.737**	**<0.001**	−935.989	0.124	**−238.122**	**0.024**
OTH	**1439.161**	**<0.001**	**−641.019**	**0.026**	**−265.996**	**<0.001**
POS	**−563.782**	**<0.001**	55.797	0.823	**−163.057**	**<0.001**
PPO	**279.395**	**0.004**	−540.616	0.096	**−220.862**	**<0.001**
**Received Surgery Post-Diagnosis**	**636.44**	**<0.001**	**829.55**	**<0.001**	−24.814	0.433
**Provider pRDI Category**						
I						
II	**276.364**	**<0.001**	97.673	0.675	19.296	0.632
III	**439.061**	**<0.001**	**849.411**	**<0.001**	17.006	0.672
**Comorbidities**						
Congestive Heart Failure	−505.04	0.096	−326.31	0.746	−122.111	0.483
Cardiac Arrhythmia	125.699	0.500	−84.224	0.891	−33.675	0.752
Valvular Disease	220.38	0.464	−172.757	0.863	−42.285	0.806
Pulmonary Circulation Disorders	−204.489	0.665	−552.753	0.724	−40.923	0.880
Peripheral Vascular Disorders	55.874	0.826	−129.424	0.878	**530.424**	**<0.001**
Hypertension Uncomplicated	−51.218	0.595	−18.985	0.953	−102.452	0.063
Hypertension Complicated	−214.48	0.499	−124.797	0.906	−152.06	0.403
Paralysis	788.05	0.134	629.979	0.717	−118.86	0.693
Chronic Pulmonary Disease	259.933	0.063	283.419	0.541	36.887	0.646
Diabetes Uncomplicated	**688.99**	**<0.001**	125.795	0.778	−60.088	0.437
Diabetes Complicated	**517.228**	**0.042**	−215.891	0.798	**520.013**	**<0.001**
Hypothyroidism	243.271	0.159	−187.655	0.743	−79.269	0.424
Renal Failure	−466.209	0.115	255.195	0.795	−53.658	0.752
Liver Disease	−257.867	0.390	−450.065	0.650	−116.873	0.496
Peptic Ulcer Disease excluding bleeding	377.287	0.623	−1041.792	0.682	9.399	0.983
AIDS/HIV	−297.161	0.710	−1294.699	0.625	−11.993	0.979
Rheumatoid Arthritis/Collagen	419.5	0.136	377.811	0.685	**691.935**	**<0.001**
Coagulopathy	11.361	0.978	**13257.089**	**<0.001**	−24.972	0.916
Obesity	−322.404	0.240	−13.938	0.988	182.74	0.245
Weight Loss	748.606	0.072	31.574	0.982	−73.72	0.757
Fluid and Electrolyte Disorders	−191.583	0.455	−999.169	0.239	7.816	0.958
Blood Loss Anemia	−690.447	0.367	−191.622	0.940	−108.058	0.805
Deficiency Anemia	9.335	0.970	−270.483	0.745	−93.898	0.514
Alcohol Abuse	−388.13	0.529	−391.862	0.848	246.143	0.486
Drug Abuse	64.879	0.923	−539.659	0.808	−273.219	0.477
Psychoses	3.204	0.995	−285.142	0.858	67.88	0.805
Depression	−88.445	0.609	−109.414	0.849	**402.072**	**<0.001**

## Data Availability

No new data were created in this study. The data presented in this study are openly available in Optum Clinformatics Datamart Database.

## Abbreviations

American Society of Clinical Oncology (ASCO)
Central nervous system (CNS)
Current Procedural Terminology (CPT)
Exclusive Provider Organization (EPO)
Glioblastoma (GBM)
Health Maintenance Organization (HMO)
Indemnity (IND)
International Classification of Diseases (ICD) system
Other (OTH)
Palliative care (PC)
Point-of-service (POS)
Provider patient racial diversity index (provider pRDI)
